# Clinicopathological features and outcome for neuroendocrine neoplasms of gastroesophageal junction: A population‐based study

**DOI:** 10.1002/cam4.1702

**Published:** 2018-07-31

**Authors:** Panpan Zhang, Wei Wang, Ming Lu, Changqing Zeng, Jie Chen, Enxiao Li, Huangying Tan, Wei Wang, Xianjun Yu, Qiyun Tang, Jiemin Zhao, Lin Shen, Jie Li

**Affiliations:** ^1^ Beijing Cancer Hospital Peking University Cancer Hospital & Institute Beijing China; ^2^ Sun Yat‐sen University Cancer Center Guangzhou China; ^3^ Fujian Province Hospital Fujian China; ^4^ The First Affiliated Hospital of Sun Yat‐sen University Guangzhou China; ^5^ The First Affiliated Hospital of Xi'an Jiaotong University Xi'an China; ^6^ China‐Japan Friendship Hospital Beijing China; ^7^ Huadong Hospital Shanghai China; ^8^ Fudan University Shanghai Cancer Center Shanghai China; ^9^ Jiangsu Province Hospital Jiangsu China; ^10^ The First Hospital of Changzhou Jiangsu China

**Keywords:** clinicopathological characteristics, gastric, gastroesophageal junction, neuroendocrine neoplasm, SEER

## Abstract

**Background:**

Gastroesophageal Junction neuroendocrine neoplasms (GEJ‐NENs) are rare and heterogeneous tumors. We aim to analyze the clinicopathlogical features and prognostic factors of GEJ‐NENs and to compare the outcome of GEJ‐NENs with other gastric NENs.

**Methods:**

A total of 297 GEJ‐NENs patients were enrolled from 10 Chinese hospitals and 3152 gastric NENs patients, including 274 GEJ‐NENs, were retrieved from Surveillance, Epidemiology, and End Results (SEER) database.

**Results:**

The clinical characteristics of GEJ‐NENs among different races were different. All Chinese patients had GEJ‐NENs of grade 3, with 67.7% of poorly differentiated NEC and 32.3% of MANEC. In SEER database, 70.8% of white, 62.5% of black, and 87.5% of AP patients had poorly differentiated/undifferentiated tumors. In Cox multivariate analysis, NEC/MANEC (HR 2.09, 95%CI 1.24‐3.56; *P* = 0.006), lymph node metastasis (HR 3.52, 95%CI 1.68‐7.34; *P* = 0.001), and distant metastases (HR 3.90, 95%CI 2.50‐6.08; *P* < 0.001) are independent predictors of overall survival. Surgical resection showed a median OS improvement of 13.1‐73.3 months (HR 0.21, 95% CI 0.14‐0.33, *P* < 0.001). Adjuvant therapy did not improve survival for postoperative GEJ‐NEN patients (*P* = 0.141). GEJ‐NENs were larger, higher grade, more distant metastasis, and worse prognosis than other gastric NENs.

**Conclusion:**

GEJ‐NENs were mostly poorly differentiated carcinomas, and all of Chinese patients were NEC/MANEC. The outcome of MANEC was preferable to NECs. Both lymph nodes metastasis and distant disease were independent predictors of prognosis. Surgical resection can improve survival, but postoperative adjuvant therapy had no additional benefit. GEJ‐NENs have worse survival than other gastric NENs.

## 1 INTRODUCTION

Neuroendocrine neoplasm (NEN) presents a heterogeneous group of tumors arising from neuroendocrine cells of the diffuse neuroendocrine system.^1^ Multiple factors may influence the outcome of the NENs, and the tumor location is one factor determined the malignancy of the tumor. Furthermore, the various incidence and characteristics of the NENs between different populations suggested a racial disparity.^2^ According to SEER database, the rectum and small intestine were the most common sites for NENs, and those in the stomach were less frequent.^2^, ^3^ Epidemiological data from both Korea and Taiwan indicate that gastric NEN is the second common site of NENs in the digestive tract.^4^, ^5^ Studies from Norway^6^ and England^7^ also revealed the incidence of gastric NENs surpassed that of small intestinal and colorectal NENs. However, the epidemiologic pattern for NENs of gastroesophageal junction has not been fully described. Although classified as similar entities in both historical and classification schemes, GEJ‐NENs behave more aggressively than those located elsewhere in the stomach. The current understanding of GEJ‐NENs is based on case reports and limited single‐institution case series.^8^, ^9^

Given the relative rarity of GEJ‐NENs, population‐based analyses are critical to provide an overview about the epidemiological and therapeutic trends for these subtypes. The primary aim of this study was to investigate the clinical and pathological features of Chinese patients with GEJ‐NEN by comparing with those from Surveillance, Epidemiology, and End Results (SEER) Cancer Registry, and to study the prognostic predictors for GEJ‐NENs using a multicenter cohort from China. A second aim was to characterize the GEJ‐NENs compared with other gastric NENs using a population‐based registry.

## 2 MATERIALS AND METHODS

Clinical data of patients with pathology confirmed GEJ‐NENs from 2000 to 2017 were retrieved from 10 hospitals in China. All of these hospitals were representative centers which located in different parts of China. This study was approved by the hospital institutional review board.

The study cohort included all patients registered in the SEER database from 2000 to 2013. Individual cases were retrieved with the SEER*Stat software (version 8.1.5, 31 March 2014; Cancer Statistics Branch, NCI, Bethesda, MD). Because of the SEER database's inclusion of unidentifiable patient information, this study was exempted for approval by the Office of Human Subjects Research of the National Institutes of Health. We identified patients with NETs using the following ICD‐O‐3 codes: 8240‐8249. 8240, carcinoid tumor; 8241, enterochromaffin; 8242, enterochromaffin‐like; 8243, goblet; 8244, mixed adenoneuroendocrine carcinoma; 8245, adenocarcinoid; 8246, neuroendocrine carcinoma; and 8249, atypical carcinoid. We selected NENs with primary of stomach (site code: C16.0‐16.9). Exclusion criteria included age less than 18 years, NEN as the second primary malignancy, NENs diagnosed at autopsy or death, and diagnoses without microscopic confirmation.

The following variables were included in the analysis: age at diagnosis, race, sex, year of diagnosis, primary tumor location, tumor grade and differentiation, AJCC staging, nodal status, distant metastasis, type of surgery performed, and OS. Tumor grade according WHO 2010 classification based on Ki‐67 index and mitotic count was analyzed from Chinese cohort.^10^ Tumor stages were assigned according to the staging classification sequentially proposed by European Neuroendocrine Tumor Society (ENETS) and American Joint Committee on Cancer (AJCC)^11^, ^12^ which were identical in NENs of stomach.

### 2.1 Statistical analysis

To investigate the clinicopathological characteristics of the study patients, Student's *t* test, χ^2^ test (or Fisher exact test) and Mann‐Whitney method were used. Overall survival (OS) time was measured from the date of initial diagnosis until the date of death or last follow‐up. Survival analysis was performed with OS as the primary outcome measure. Survival was evaluated using Kaplan‐Meier estimates and Cox proportional hazard regression. Statistical tests used two‐tailed *P* values, and *P* < 0.05 was considered statistically significant. All statistical analyses were performed in SPSS (version 25; IBM, Chicago, IL).

## 3 RESULTS

### 3.1 Clinicopathological characteristics of Chinese patients with GEJ‐NENs

We retrospectively analyzed clinical and pathologic features of 297 patients with histological confirmed GEJ‐NENs from 10 hospitals. The entire group had a median age of 63 (35‐85), and 87.2% were male (n = 259). Based on the WHO‐2010 grading classification, all the patients with GEJ‐NENs were grade 3, and the proportion of poorly‐differentiated NEC and MANEC was 67.7% and 32.3%. Regional lymph node metastasis was found in 155 (52.2%) patients and 78 (26.2%) had distant metastasis at diagnosis. According to the AJCC/UICC staging system, 2 (0.7%) patients were classified as stage I, 46 (15.5%) as stage II, 171 (57.6%) as stage III and the other 78 (26.2%) as stage IV, respectively.

Compared with their NEC counterparts, the MANECs were more highly associated with early stage (*P* = 0.002), curative operations (90.6% vs. 71.1%, *P* < 0.000), and more lymphatic metastasis (60.4% vs. 48.3%, *P* < 0.000), but were less associated with distant metastasis (14.6% vs. 31.8%, *P* < 0.000). The MANEC and NEC groups were statistically similar in other clinicopathological characteristics, including gender, age, Ki67 index, and tumor size. The comparison of clinicopathological characteristics between NEC and MANEC of the gastroesophageal junction is shown in table 1.

**Table 1:** Comparison of clinical features of NEC and MANEC in Chinese GEJ‐NENs

Characteristics	NEC(n = 201)	MANEC(n = 96)	*P* value
Age median, years	63.1 ± 8.6	62.1 ± 9.1	0.297
Male, n (%)	175 (87.1%)	83 (86.5%)	0.885
Ki67 index, %	69.1 ± 17.1	66.7 ± 17.9	0.717
Tumor size, cm	4.7 ± 1.9	4.6 ± 1.8	0.907
Surgery, n (%)	143 (71.1%)	87 (90.6%)	<0.000
AJCC Stage			
I‐II	25 (12.4%)	23 (24.0%)	0.002
III‐IV	176 (87.6%)	73 (76.0%)	
Regional lymph nodes, n (%)	97 (48.3%)	58 (60.4%)	<0.000
Distant metastasis, n (%)	64 (31.8%)	14 (14.6%)	<0.000

### 3.2 Comparison of the clinicopathological characteristics of GEJ‐NENs in different races

In total, 297 and 274 patients with GEJ‐NENs were included, respectively, from the Chinese cohort and SEER database. The clinicopathological characteristics of GEJ‐NENs among different races were distinct. The mean ages were 62.7, 64.5, 58.5, and 61.5, respectively, in Chinese, white, black patients, and Asian/Pacific Islander (AP) patients. Except for black patients, male patients were more frequent. In Chinese patients, tumor size was larger than that in other groups. Chinese patients were poorly differentiated NEC and MANEC. In SEER database, 70.8% of white, 62.5% of black and 87.5% of AP patients had poorly differentiated/undifferentiated tumors. Distant metastasis at the time of presentation was more frequent in white and black patients than Chinese patients (60.0% vs.55.6% vs.26.3%, *P* < 0.000). Surgical of primary tumor was performed in most of the patients in different race groups. Table 2 summarizes baseline characteristics of GEJ‐NENs among different races.

**Table 2:** Comparison of the clinicopathological characteristics of GEJ‐NENs among different races

		SEER database			
Characteristics	Chinese patients (n = 297)	White patients (n = 226)	Black patients (n = 32)	Asian/Pacific Islander patients (n = 16)	*P* value
Age, y					
Median,95%CI	62.7 (61.7‐63.8)	64.5 (62.8‐66.2)	58.5 (53.5‐63.5)	61.5 (55.2‐67.8)	<0.000
Sex					
Male, n (%)	258 (86.9)	137 (60.6)	15 (46.9)	9 (56.3)	<0.000
Female, n (%)	39 (13.1)	89 (39.4)	17 (53.1)	7 (43.8)	
Size, cm					
Mean,95%CI	4.7 (4.4‐4.9)	3.0 (2.5‐4.1)	3.1 (1.8‐4.4)	3.8 (1.7‐7.1)	<0.000
Range	0.3‐12	0.1‐15	0.4‐8.5	0.4‐10.6	
Morphology					
NEC	201 (67.7)				‐
MANEC	96 (32.3)				
Grade					
Well/Moderate differentiated		42 (29.2)	6 (27.5)	1 (12.5)	<0.000
Poorly/undifferentiated		102 (70.8)	10 (62.5)	7 (87.5)	
AJCC Stage					
I‐II	48 (16.2)	40 (27.6)	7 (38.9)	3 (37.5)	<0.000
III	171 (57.6)	18 (12.4)	1 (5.6)	3 (27.5)	
IV	78 (26.3)	87 (60.0)	10 (55.6)	2 (25.0)	
Surgery, n (%)					
performed	230 (77.4)	81 (36.5)	16 (50.0)	9 (56.3)	<0.000
Unperformed	67 (22.6)	141 (63.5)	16 (50.0)	7 (43.8)	

### 3.3 Treatment and survival of Chinese patients with GEJ‐NENs

**Figure 1:**
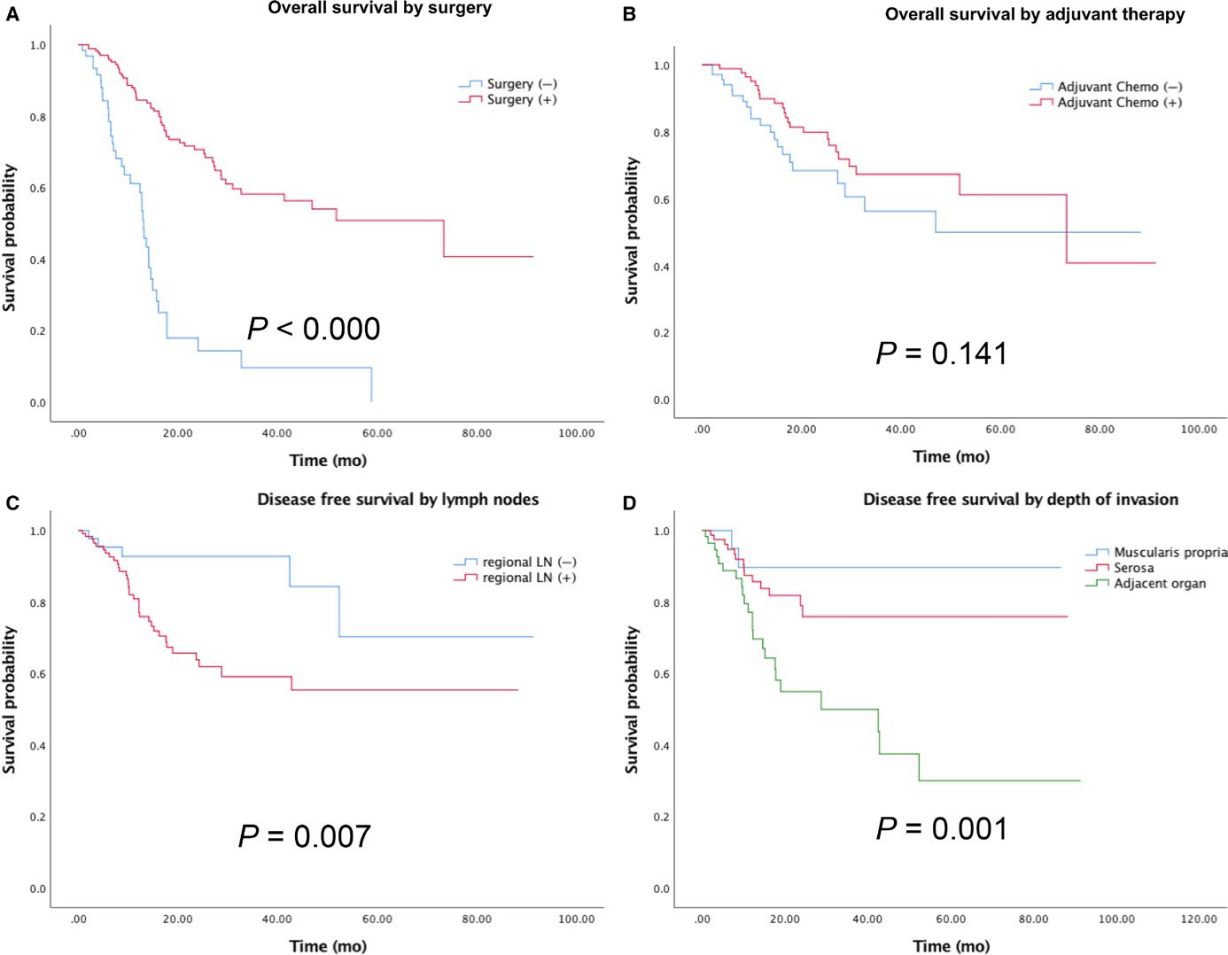
Overall survival (A) by surgery resection; (B) by adjuvant treatment; Disease free survival (C) by regional lymph nodes metastasis; (D) by invasion depth

Overall, treatment strategies were provided in all the 297 patients. Among these patients, 77.4% (n = 230) underwent surgical resection, of which 89.6% (n = 206) were curative and 10.4% (n = 24) were palliative. Surgical resection offered a survival advantage with HR 0.21 (95%CI 0.14‐0.33) and the median OS improved from 13.1 to 73.3 months (*P* < 0.001) (Figure 1A). A total of 104 (50.5%) patients received adjuvant chemotherapy. There was no additional survival benefit to adjuvant chemotherapy in patients undergoing surgical resection (*P* = 0.141) (Figure 1B). Invasion depth, lymph node metastasis can predict the risk of postoperative recurrence (Figure 1C,D).

**Figure 2:**
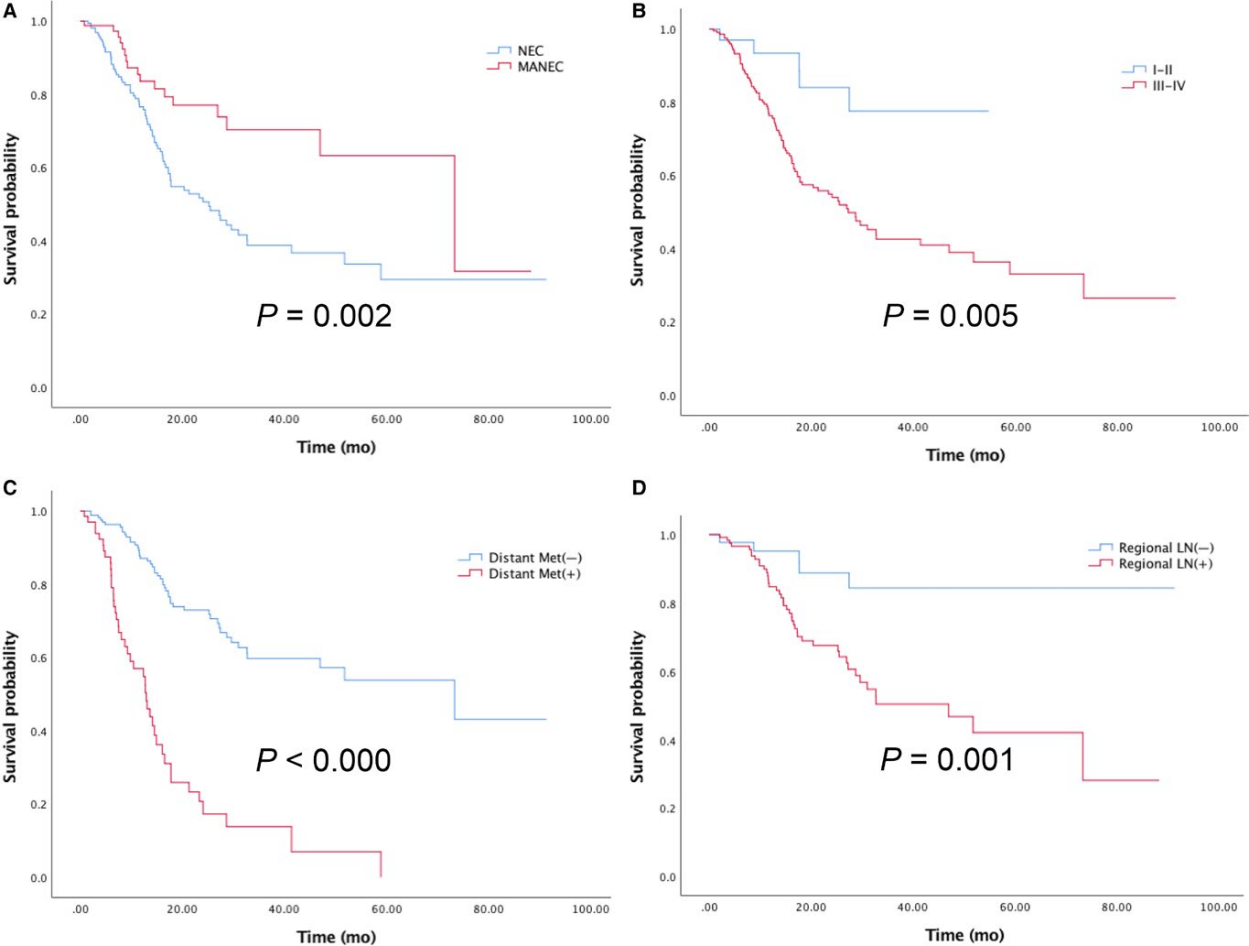
Overall survival (A) by NEC vs. MANEC; (B) by stage; (C) by distant metastasis; (D) by regional lymph nodes status

The median survival time of the entire GEJ‐NENs patients was 31.0 months (95% CI 16.6‐45.4mo), and the subgroup of MANEC had longer survival than NEC (73.3 vs. 25.2, *P* = 0.002). On multivariate analysis, NEC/MANEC (HR 2.09, 95%CI 1.24‐3.56; *P* = 0.006) (Figure 2A), stage (HR 3.29,95%CI 1.33‐8.12; *P* = 0.010) (Figure 2B), lymph nodes metastasis (HR 3.52,95%CI 1.68‐7.34; *P* = 0.001) (Figure 2C), and distant metastases (HR 3.90,95%CI 2.50‐6.08; *P* < 0.001) (Figure 2D) were independent predictors of overall survival (Table 3).

**Table 3:** Univariate and multivariate analysis of characteristics predicting overall survival

	Univariate cox regression			Multivariate cox regression		
	*P* value	HR	95% CI	*P* value	HR	95% CI
Age						
<65y	‐	1				
≥65y	0.36	1.21	0.80‐1.81			
Size						
<4.5 cm	‐	1				
≥4.5 cm	0.007	2.37	1.85‐3.92			
Sex						
Female	‐	1				
Male	0.31	1.32	0.77‐2.26			
Ki67 index						
<70%	‐	1				
≥70%	0.48	1.17	0.74‐1.83			
Morphology						
Large cell	‐	1				
Small cell	0.75	1.12	0.56‐2.23			
MANEC/NEC						
MANEC	‐	1		‐		
NEC	0.002	2.25	1.33‐3.81	0.006	2.09	1.24‐3.56
Stage						
I‐II	‐	1		‐	1	
III‐IV	0.005	3.61	1.46‐8.89	0.010	3.29	1.33‐8.12
Lymph nodes						
No	‐	1			1	
Yes	0.000	4.15	2.01‐8.61	0.001	3.52	1.68‐7.34
Metastasis						
No	‐	1		‐	1	
Yes	0.000	4.89	3.21‐7.46	0.000	3.90	2.50‐6.08

### 3.4 Comparison of GEJ‐NEN and non‐GEJ NENs of SEER database

A total of 3152 patients with gastric NENs were identified in the SEER database from 2000 to 2013, including 274 GEJ‐NEN patients and 2878 non‐GEJ NEN patients. GEJ‐NENs were more commonly diagnosed at an older age (63.6 vs. 62.9 years, *P* < 0.000) and in white patients (82.5% vs. 78.4%, *P* < 0.000). Patients with GEJ‐NEN were more frequently male than female (58.5% vs. 40.1%, *P* < 0.001). Tumors of GEJ‐NENs were larger (3.0 vs. 1.9 cm, *P* < 0.000) and predominantly poorly differentiated and undifferentiated tumors (70.8% vs.19.5%, *P* < 0.001). GEJ‐NENs were highly invasive with more distant metastases (41.5% vs.11.9%, *P* < 0.001), whereas non‐GEJ NENs had more localized lesions (79.5% vs. 43.6%, *P* < 0.000). Patients with GEJ‐NENs were less likely to be treated with resection (39.3% vs. 59.9%, *P* < 0.000), but more likely to receive radiotherapy (6.2% vs.2.1%, *P* < 0.001). Table 4 contains patient characteristics that were assessed between GEJ‐NENs and other gastric NENs.

**Table 4:** Clinical and pathological features of GEJ and other gastric NEN in the SEER database

Variable	GEJ‐NEN(n = 274)	Non‐GEJ NEN(n = 2878)	*P* value
Age, median, years	63.6 ± 13.1	62.9 ± 13.6	<0.000
Male, n (%)	161 (58.5%)	1155 (40.1%)	<0.000
Race			
White	226 (82.5%)	2257 (78.4%)	<0.000
Black	32 (11.7%)	385 (13.4%)	
Asian	15 (5.5%)	174 (6.0%)	
Other	1 (0.4%)	62 (2.2%)	
Grade			
Well/Moderate differentiated	49 (29.25%)	1022 (80.5%)	<0.000
Poorly/undifferentiated	119 (70.8%)	248 (19.5%)	
ICD‐O‐3 Code			
Carcinoid (8240‐8243,8249)	99 (36.1%)	2061 (71.6%)	<0.000
Neuroendocrine carcinoma (8246)	172 (62.8%)	777 (27.0%)	
Mixed adenoneuroendocrine (8244)	2 (0.7%)	25 (0.9%)	
Adenocarcinoma (8245)	1 (0.4%)	15 (0.5%)	
Tumor size, cm	3.0 (0.1‐15.0)	1.9 (0.1‐12.0)	<0.000
Surgery, n (%)	106 (39.3%)	1697 (59.9%)	<0.000
Radiation, n (%)	44 (6.2%)	60 (2.1%)	<0.000
AJCC Stage			
I‐II	50 (29.1%)	1274 (75.6%)	<0.000
III‐IV	121 (70.8%)	411 (24.4%)	
Extent of disease			
Local	105 (43.6%)	1878 (79.5%)	<0.000
Regional	236 (14.9%)	204 (7.6%)	
Distant	100 (41.5%)	281 (11.9%)	

ICD‐O‐3, International Classification of Diseases for Oncology, 3rd Edition.

**Figure 3:**
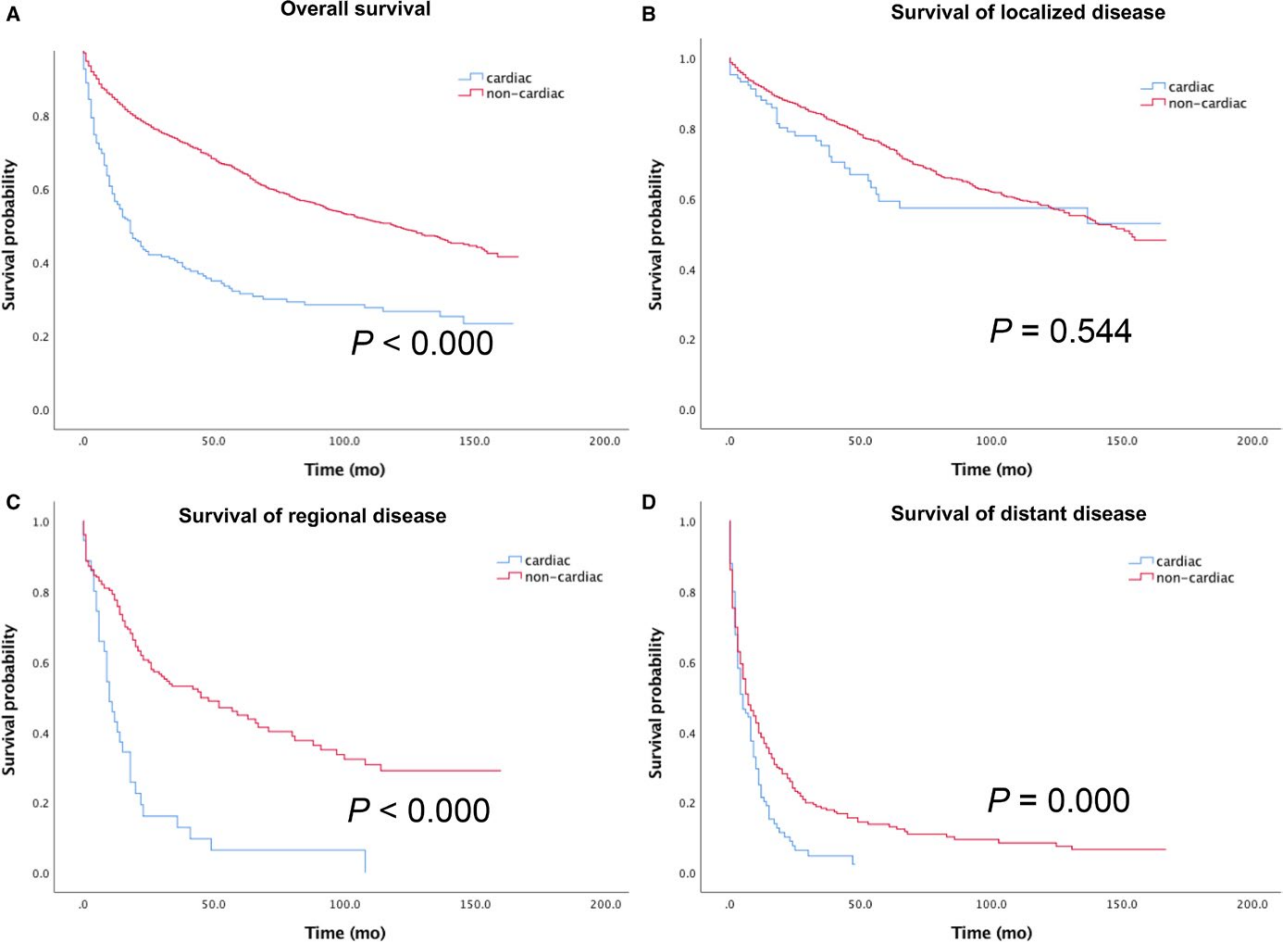
Overall survival of (A) the entire gastric NENs cohort and for the subgroup of (B) localized disease, (C) regional disease, (D) distant disease

Median OS was significantly worse for patients with GEJ‐NENs than those with non‐GEJ NENs (*P* < 0.000) (Figure 3A). Stratified analysis by stage showed that: in localized disease, median survival showed no difference (Figure 3B); in regional or metastatic disease, median survival of GEJ‐NENs showed worse survival (*P* < 0.001) (Figure 3C&D).

## 4 DISCUSSION

Gastroesophageal NENs account for a relatively small and heterogeneous population with an aggressive course; however, limited information is available regarding characteristics because of its rarity.^13^, ^14^ In this study, we investigated the clinical characteristics and outcomes of patients diagnosed with GEJ‐NENs using a population‐based registry from 10 Chinese hospitals in China and the SEER database. This study represents one of the largest and most detailed cohort analyses of the epidemiology and outcomes of GEJ‐NENs. We found the clinicopathologic features of GEJ‐NENs differ among different races, and Chinese patients mostly had poorly differentiated disease, including relatively high‐frequency of MANEC. Additionally, we showed that the GEJ‐NENs were particularly more aggressive than other gastric NENs.

The current study indicated that all the Chinese patients with GEJ‐NENs were poorly differentiated carcinomas, and 96 cases (32.3%) were MANEC with both adenocarcinoma and neuroendocrine differentiation. According to gastric NENs clinical classification,^15^ GEJ‐NENs are prone to be sporadic and poorly differentiated and thus classified as the fourth subtype. GEJ‐NENs were male dominated and gross appearance of the tumor was large in size. Due to aggressive biological behavior, GEJ‐NENs frequently metastasized to regional lymph nodes and distant organs and thus had a poor prognosis. Half of the patients had regional lymph node metastasis and one‐third of patients showed distant metastasis. For such heterogeneous carcinoma with aggressive behavior, multidisciplinary team is recommended during the process of clinical management and medical care.

Because inadequate understanding of GEJ‐NENs, it is controversial of the prognostic value of their histologic classification. Compared with the adenocarcinoma, NEC was more aggressive with poorly differentiated morphology.^16^ Shia et al reported^17^ the absence of an associated adenocarcinoma component was predictive of a worse outcome; however, previous studies about gastric or colorectal MANEC reported that there was no statistically significant difference in survival between MANECs and NEC.^18^, ^19^ In our cohort, a number of GEJ‐NECs were mixed with high grade adenocarcinoma, the outcome of which was better than pure NECs. Then we compared the clinicopathological features between MANEC and NEC, and there was no difference in age, sex, Ki67 index, and tumor size. However, metastatic patterns of the two entities were different: The regional lymph node metastasis of MANEC was more common, and distant metastasis frequently occurred in NEC, indicating that the behavior of NEC may be more aggressive.

The importance of surgical resection of gastric NENs on survival has been reported in the previous literature.^20^, ^21^ Accordingly, a wide spectrum of therapeutic options has been provided, from endoscopic follow‐up to curative partial or total gastrectomy.^22^ In our study, GEJ‐NENs mostly were diagnosed at stage III‐IV, and after surgery recurrence occurred in a relatively large number of patients. But it remains controversial whether adjuvant therapy reduces the risk of recurrence or prolongs the overall survival of GEJ‐NENs. In Chinese cohort, 104 (50.5%) patients received platinum‐based (cisplatin or oxaliplatin) adjuvant chemotherapy after radical surgery, but the cohort received adjuvant therapy showed no additional survival advantage. We should be cautious about drawing the conclusion as the adjuvant regimens included protocols referring to gastric adenocarcinoma and neuroendocrine carcinoma. Therefore, the role of adjuvant chemotherapy after radical resection and the specific regimen to choose need further investigation.

Although classified as similar entities in classification schemes, tumors of gastroesophageal have distinct characteristics compared with those located elsewhere in the stomach, which has been observed in gastric adenocarcinoma.^23^ Previous studies indicated that GEJ‐NENs were more similar to esophageal NENs than to those of the stomach, with more aggressive behavior.^24^ We compared the characteristics and outcome of GEJ‐NENs with other gastric NENs using SEER database, and the result showed that patients with GEJ‐NENs had tumors larger in size. The majority of the cohort was diagnosed at more advanced stage and tends to be poorly differentiated compared with non‐GEJ NENs entities. The unfavorable prognosis also indicated that GEJ‐NENs were more aggressive than their counterparties located in other sites of the stomach. Therefore, the morphology and histology may be a potential cause for the worse survival of GEJ‐NENs.

It has been reported that the biological behavior and clinical outcome of patients with gastric carcinoma varies among different human races.^25^ In our study, by comparing the clinicopathologic characteristics of GEJ‐NENs among different races, large disparities were found in terms of histology grade and clinical stage; The Chinese cohort of GEJ‐NENs is high‐grade MANE/NEC, while Asian/Pacific Islanders (AP) patients from SEER database had more poorly differentiated/undifferentiated tumors than white and black patients. Moreover, tumor size of Chinese patients was significantly larger than that in other groups of patients. Upon diagnosis, distant metastasis was less common in Chinese patients than that in white and black patients from the SEER database. This situation was also found in AP patients with GEJ‐NENs from the SEER database. Therefore, different genetic and epigenetic changes may partly explain the diversity among different races.

It is acknowledged that this study has limitations. The primary limitation is that the SEER database does not include information of Ki‐67 index and data on recurrence or disease‐free survival. The chemotherapy and specific treatment regimens were not included. The second limitation is the retrospective nature of our study. The pathological data from achieves were according to the 2010 WHO classification, although a new term of mixed neuroendocrine‐non‐neuroendocrine neoplasm(MiNEN) was proposed, considering the morphological and biological heterogeneity of gastrointestinal mixed tumors.^26^ However, all the MANECs in our entity are consistently poorly differentiated neuroendocrine carcinoma combined with adenocarcinoma. Moreover, we include 298 Chinese patients with GEJ‐NEN and a large sample of GEJ‐NEN patients from the SEER database. The cohort in our study represents the largest dataset of GEJ‐NENs to date and offers valuable information on the epidemiology and prognosis. This study provides a uniquely detailed assessment of the GEJ‐NENs to improve our understanding of the disease and guide future research.

## 5 CONCLUSIONS

GEJ‐NENs were highly invasive with frequent distant metastases, with predominantly poorly differentiated and undifferentiated tumors, thus showed worse survival than other gastric NENs. The clinicopathological characteristics of GEJ‐NENs among different races were distinct. Chinese patients with GEJ‐NENs are all NEC or MANEC, and the latter show distinct metastatic patterns and better survival. Surgical resection improved the survival, and there was no additional survival benefit to adjuvant chemotherapy, and prospective studies using defined diagnostic criteria are necessary to determine optimal management.

## CONFLICT OF INTEREST

None declared.
